# Polymeric and Solid Lipid Nanoparticles for Sustained Release of Carbendazim and Tebuconazole in Agricultural Applications

**DOI:** 10.1038/srep13809

**Published:** 2015-09-08

**Authors:** Estefânia Vangelie Ramos Campos, Jhones Luiz de Oliveira, Camila Morais Gonçalves da Silva, Mônica Pascoli, Tatiane Pasquoto, Renata Lima, P. C. Abhilash, Leonardo Fernandes Fraceto

**Affiliations:** 1 Department of Environmental Engineering, State University of São Paulo (UNESP), Sorocaba, SP, Brazil; 2 Department of Biochemistry, Institute of Biology, State University of Campinas (UNICAMP), Cidade Universitária Zeferino Vaz, Campinas, SP, Brazil; 3 Department of Biotechnology, University of Sorocaba, Sorocaba, SP, Brazil; 4 Institute of Environment & Sustainable Development, Banaras Hindu University, Varanasi, India

## Abstract

Carbendazim (MBC) (methyl-2-benzimidazole carbamate) and tebuconazole (TBZ) ((RS)-1-(4-chlorophenyl)-4,4-dimethyl-3-(1H-1,2,4-triazol-1-ylmethyl)pentan-3-ol) are widely used in agriculture for the prevention and control of fungal diseases. Solid lipid nanoparticles and polymeric nanocapsules are carrier systems that offer advantages including changes in the release profiles of bioactive compounds and their transfer to the site of action, reduced losses due to leaching or degradation, and decreased toxicity in the environment and humans. The objective of this study was to prepare these two types of nanoparticle as carrier systems for a combination of TBZ and MBC, and then investigate the release profiles of the fungicides as well as the stabilities and cytotoxicities of the formulations. Both nanoparticle systems presented high association efficiency (>99%), indicating good interaction between the fungicides and the nanoparticles. The release profiles of MBC and TBZ were modified when the compounds were loaded in the nanoparticles, and cytotoxicity assays showed that encapsulation of the fungicides decreased their toxicity. These fungicide systems offer new options for the treatment and prevention of fungal diseases in plants.

The fungal diseases that affect crops worldwide are not a new problem. In the 19^th^ century, a fungal disease destroyed potato crops in Ireland and led to one of the greatest European famines of those times. The damage caused by fungi in five of the world’s most important crop plants (rice, wheat, maize, potato, and soybean) has an estimated annual cost of more than $60 billion, and the effective control of fungal diseases would result in an increase in food production equivalent to the ability to feed more than 600 million persons annually^1^,^2^.

Carbendazim (MBC) (methyl-2-benzimidazole carbamate) is a systemic benzimidazole fungicide and its mode of action consists of the inhibition of cellular division. MBC shows low solubility in water (8 mg/L at 25 °C) and a pKa of 4.48. This compound possesses a benzimidazolic ring that is hard to break, so degradation occurs very slowly and MBC may persist for a long time in the environment^3^,^4^. Tebuconazole (TBZ) ((RS)-1-(4-chlorophenyl)-4,4-dimethyl-3-(1H-1,2,4-triazol-1-ylmethyl)pentan-3-ol) is a triazole class systemic fungicide with a broad spectrum of action. The fungicides of this class prevent fungal growth by inhibiting the biosynthesis of ergosterol. TBZ also shows low aqueous solubility (36 mg/L at 25 °C). The pKa of TBZ has not yet been determined because the compound is a very weak base^2^,^5^,^6^,^7^. According to Montuelle *et al*.^8^, tebuconazole is increasingly present in stream water. This fungicide is toxic to organisms and may cause adverse effects following chronic exposure in the aquatic environment^9^. The concentrations of TBZ in surface water can be up to 175–200 μg/L^10^.

Systemic fungicides are usually highly selective because they need to penetrate the vascular system of the plant in order to reach the invading fungus. Due to the specific modes of action of these fungicides, their effectiveness can be negated even by small genetic modifications in fungi, and repeated applications can become ineffective. One way of reducing the risk of resistance as well as increasing the effectiveness of fungicides against pathogens, is to produce formulations that include fungicides from different chemical classes^11^,^12^. These types of product can confer the management of resistance to fungicides by combining chemicals that exhibit distinct mechanisms of action. Nonetheless, despite the use of combinations of fungicides, the critical factors such as photodegradation, volatilization, and leaching can reduce the biological activity of the active agents, so that more applications are required to achieve the desired effect. In turn, this can cause greater impacts in the environment and on human health^13^.

In recent years, there have been major efforts to develop carrier systems for pesticides that are able to modify the release profiles and increase the effectiveness of the formulations for the effective control of agricultural pests^13^,^14^. Polymeric systems have attracted the attention due to their ability to provide sustained release of the associated active compounds. For example, poly(ε-caprolactone) is an aliphatic polyester that is insoluble in water, biodegradable, and biocompatible, amongst other useful physicochemical properties^15^,^16^. This polymer can be used to synthesize the nanocapsules possessing an oily interior that are capable of efficiently encapsulating hydrophobic compounds including tebuconazole and carbendazim^17^,^18^,^19^. Solid lipid nanoparticles (SLNs) are other promising carrier systems are that can be used to transport nonpolar substances whose mobility is restricted by interaction with the lipids, resulting in modified release profiles^20^,^21^. Advantages of these carrier systems include the requirement for smaller quantities of active agents, reduced losses due to leaching, degradation, and volatilization, and lower environmental impacts^22^,^23^,^24^,^25^. The main advantages of SLNs and polymeric nanoparticles for use in agriculture are their low toxicities, since the matrices are composed of low toxicity polymers (such as poly(ε-caprolactone)) and lipids that are present in many organisms^26^,^27^. Carrier systems that have been reported for fungicides include hydrogels and spheres used as carriers for thiram^28^,^29^,^30^,^31^, silica nanospheres containing tebuconazol^13^, cyclodextrins encapsulating carbendazim^32^,^33^, and polymeric microparticles containing tebuconazole^34^.

The objective of the present study was to prepare and characterize polymeric nanocapsules and solid lipid nanoparticles that were then used as carriers for a mixture of carbendazim and tebuconazole. The nanoparticles were characterized in terms of particle size, zeta potential, and polydispersivity index. The particle morphology was analyzed using transmission electron microscopy (TEM). The encapsulation efficiencies and release profiles of the fungicides were determined *in vitro*, and the cytotoxicities of the formulations were evaluated. The effects of the formulations on the germination of beans were also determined. There have been no previous reports in the literature concerning the combination of these two fungicides in a single carrier system. Moreover, in agricultural applications, the techniques developed offer the possibility of reducing adverse effects in ecosystems, as well as diminishing risks to human health.

## Results and Discussion

**Figure 1:**
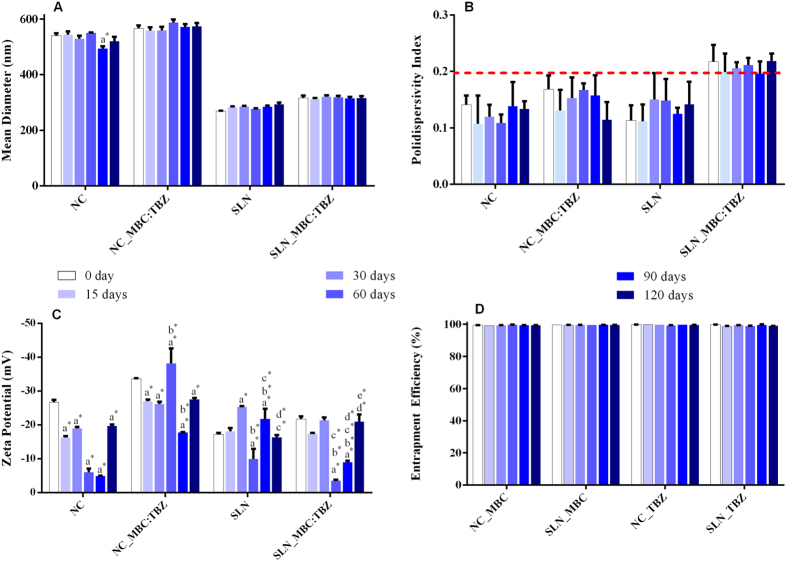
Determination of nanoparticle stability: (A) nanoparticle diameter (z-average, nm); (B) polydispersivity index; (C) zeta potential (mV); (D) encapsulation efficiency. Measurements were made of the formulations containing the polymeric nanocapsules and solid lipid nanoparticles, with and without the fungicides, at ambient temperature after different periods of storage (0, 15, 30, 60, 90, and 120 days). The values shown represent the averages of three determinations. The level of significance was p < 0.05 for the statistical differences observed between the groups, where the a* represents statistical differences compared to 0 day; b* compared to 15 days; c* for the 30 days; d* for the 60 days and e* for the 90 days.

The stability of the formulations was evaluated using measurements of average diameter, polydispersivity index, zeta potential, and encapsulation efficiency. These are the essential parameters used to obtain information concerning the nature of colloidal systems. The analyses were performed immediately after preparation and then after 15, 30, 60, 90, and 120 days of storage at ambient temperature (25 °C). The results obtained are presented in Fig. 1.

The initial hydrodynamic diameter (Fig. 1A) of the NCs without fungicides (520 nm) showed little variation over the period of the trial (120 days). The NCs containing fungicides showed an initial average diameter of 542 nm, followed by a small decrease to 479 nm after 90 days, after which the size remained stable up to 120 days, with all analyses showing a monomodal size distribution that was indicative of a only a single type of particle^35^. The average diameter of the SLNs remained virtually unchanged over 120 days, indicating that there was no tendency to form aggregates (which would have been evidenced by an increase in particle size over time). The SLNs containing the fungicides presented a greater average diameter, compared to the particles without fungicides, suggesting that reorganization of the particles occurred following incorporation of the fungicides.

The NTA measurements of the size distributions showed that for the NCs without fungicides, the particle concentration was 9.5 ± 0.4 × 10^13^ particles/mL and the hydrodynamic diameter was 677.5 ± 18.6 nm. In the case of the NCs containing fungicides, the concentration was 9.97 ± 0.6 × 10^13^ particles/mL and the hydrodynamic diameter was 713.0 ± 1.1 nm. For the SLNs without fungicides, the concentration was 7.76 ± 0.26 × 10^13^ particles/mL and the hydrodynamic diameter was 311.9 ± 15.2 nm. The SLNs with fungicides showed a concentration of 10.4 ± 0.4 × 10^13^ particles/mL and a hydrodynamic diameter of 317.6 ± 2.7 nm.

The values obtained for the polydispersivity index, which provides an indication of the size distribution of the particles, are shown in Fig. 1B. The changes in polydispersivity indices of the NCs (with and without fungicides) over time indicate the reorganization of the nanoparticle size distribution, although the values never exceeded 0.2. The SLNs containing the fungicides presented polydispersivity indices greater than 0.2, in contrast to the behavior shown by the SLNs without the fungicides. This could be explained by the incorporation of the fungicides in the nanoparticles, which increased the size distribution and consequently increased the polydispersivity index value. After 120 days, the values decreased to <0.2, probably due to the rearrangements of the particles during the storage period.

The zeta potential is a measure of the surface charge on nanoparticles, and high values are indicative of the colloidal stability. However, in the present work, the zeta potential cannot be used as a measure of stability due to the use of PVA; this surfactant exerts a steric stabilization effect, as a result the surface electrostatic repulsion cannot be considered as the main factor influencing the colloidal stability of the particles^36^,^37^,^38^. Notwithstanding, the zeta potentials (Fig. 1C) reflected the main characteristics of the particles and were negative for all the systems analyzed. The zeta potentials were not affected by the presence of the fungicides, suggesting that the latter were mainly present within the interior of the nanoparticles (rather than on the surfaces).

Measurements of the encapsulation efficiency were used to obtain a better understanding of the interaction between the fungicides and the nanoparticles. In the present study, both of the systems investigated (NCs and SLNs) are suitable carriers for hydrophobic compounds such as MBC and TBZ as it exhibits low solubility in water (8 and 36 mg/L at 25 °C, respectively)^19^ and therefore present greater affinity for the oily nucleus of the NCs and the lipid matrix of the SLNs. As a result, both fungicides showed high encapsulation efficiencies (>99%) that remained practically constant during 120 days, providing further evidence of the high affinity of the nanocarriers for the encapsulated fungicides. Similar results were reported by Asrar *et al*.^34^, those developed polymeric microparticles as carriers for TBZ and obtained encapsulation efficiencies of between 95.2 and 98.3%, as well as slow release of the active agent. Yang *et al*.^39^ developed polymeric microparticles that showed both high encapsulation efficiency (90%) and release of less than 50% of the active principle from the interior of the particles over a period of two months.

**Figure 2:**
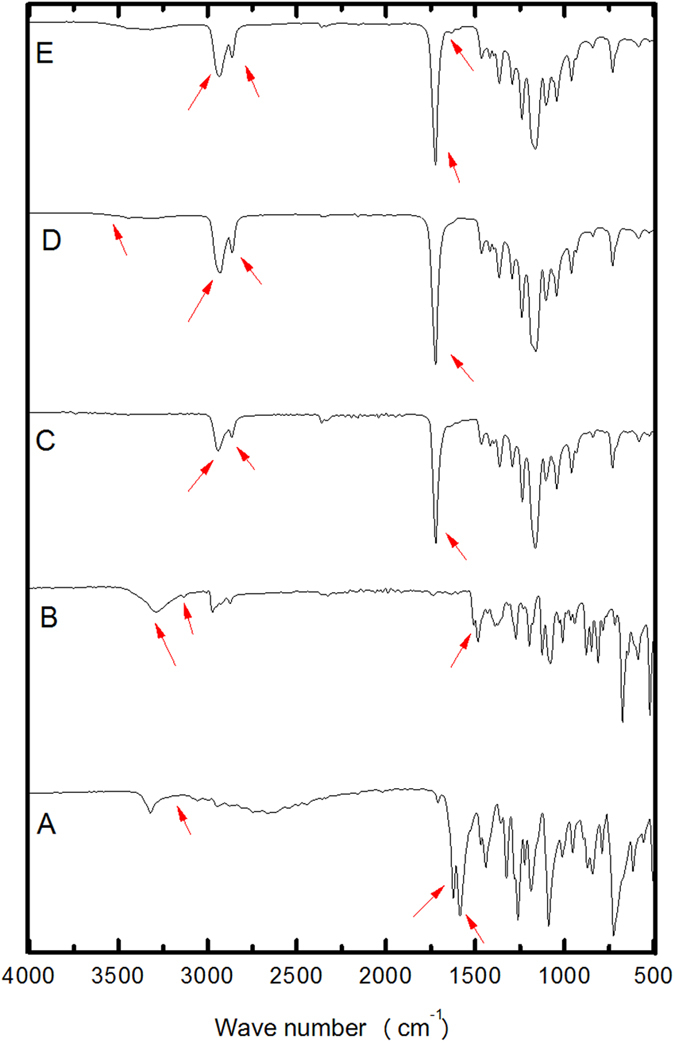
Evaluation of interaction between the fungicides and components of the PCL nanocapsule formulation. FTIR spectra for: (**A**) MBC, (**B**) TBZ, (**C**) PCL, (**D**) NC-PCL, (**E**) NC-MBC:TBZ. The arrows indicate the main characteristic absorption bands in each spectrum.

**Figure 3:**
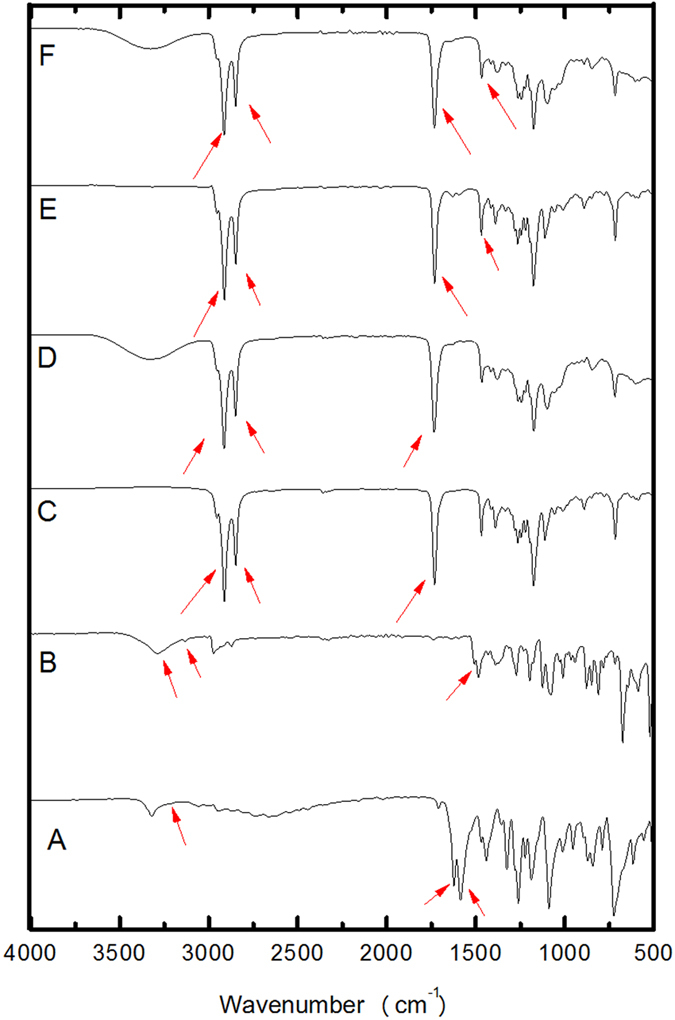
Evaluation of interaction between the fungicides and components of the SLN formulation. FTIR spectra for: (**A**) MBC, (**B**) TBZ, (**C**) tripalmitin, (**D**) physical mixture, (**E**) SLN, (**F**) SLN-MBC:TBZ. The arrows indicate the main characteristic absorption bands in each spectrum.

In order to characterize these systems FTIR was used to identify possible interactions between the fungicides and the nanoparticles, as well as to determine whether the components of the formulations were altered in any way during the preparation process. Figures 2 and 3 show the spectra for the different samples. The spectrum for MBC (Fig. 2A) revealed characteristic bands including aromatic C=C stretching at 1595 cm^−1^, stretching of ester C=O at 1630 cm^−1^, and stretching of benzene ring C−H at 3056 cm^−1^. The spectrum for TBZ (Fig. 2B) showed stretching vibration of benzene ring C−H at 3042 cm^−1^, vibration of benzene ring C=C bonds between 1570 and 1511 cm^−1^, and a band at 3300 cm^−1^ related to the alcohol group of the TBZ molecule. The PCL polymer (Fig. 2C) showed characteristic bands including the stretching of ester carbonyls (C=O) at 1735 cm^−1^ and O−H at 3440 cm^−1^, as well as C−H stretching of saturated carbon present in the polymeric chains of PCL between 3000 and 2800 cm^−1^. The spectrum for the nanoparticles without fungicides (Fig. 2D) showed that the bands remained unaltered compared to the spectrum for the PCL polymer. However, in the case of the nanoparticles containing the fungicides (Fig. 2E), in addition to the characteristic bands of the PCL polymer, there was a band related to the stretching of C=C of the aromatic ring of carbendazim, which was not observed in the spectrum of the NCs alone. However, no band corresponding to C=C stretching of the aromatic ring of tebuconazole was observed, probably due to the overlap with the carbonyl band of the PCL polymer.

The spectra for MBC, TBZ, and components of the SLN formulation are shown in Fig. 3. The spectrum for tripalmitin (Fig. 3C) showed C−H stretching bands between 2851 and 2919 cm^−1^, and stretching of C=O and C−O at 1735 cm^−1^ and 1473 cm^−1^, respectively. Superimposition of the bands corresponding to the lipid and the fungicides was observed in the spectrum for the physical mixture (Fig. 3D). The spectrum for the nanoparticles without fungicides (Fig. 3E) showed no differences, compared to the spectrum for free tripalmitin. In the case of the SLNs containing fungicides, the spectrum showed the same absorption bands corresponding to tripalmitin; no fungicide absorption bands were observed, which can be explained by the low concentrations of MBC and TBZ in the formulation, relative to the amount of lipid. Similar results were found in the literature for analysis of interaction between nanocarriers systems and encapsulated active ingredient by FTIR^40^,^41^,^42^.

**Figure 4:**
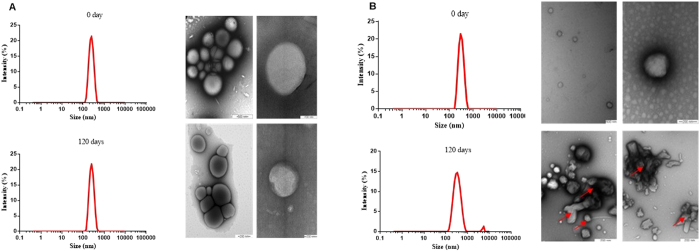
Morphological (TEM) and size distribution (PCS) analysis of the nanoparticles containing MBC and TBZ, performed immediately after preparation and then after 120 days of storage. (**A**) NC-PCL, using TEM magnifications of 27,800x and 15,600x (Day 0) and 129,300 and 21,560x (Day 120); (**B**) SLN, using TEM magnifications of 77,500x and 27,800x (Day 0) and 21,600x and 89,230x (Day 120).

Morphological characterization of the prepared nanoparticles was also done in the present study. For this, TEM was employed to investigate the morphology of the two types of nanoparticle. The NCs and SLNs were both spherical (Fig. 4) and the average particle sizes were 490 and 191.6 nm, respectively (Fig. 4A,B). The agglomerates visible in the micrographs resulted from the sample drying process. These sizes were smaller than those determined using photon correlation spectroscopy, which can be explained by the fact that the particles observed by TEM were dehydrated, while the PCS technique measured particles present in an aqueous medium.

Interestingly, the morphology of the NCs (with an average size of 498 nm) remained unchanged (Fig. 4A) even after 120 days of storage at ambient temperature. The micrograph obtained for the SLNs (Fig. 4B) revealed the presence of several nanocrystals (indicated with an arrow), which were probably composed of the fungicides. This provides a possible explanation for the second particle population observed in the size distribution obtained using DLS.

**Figure 5:**
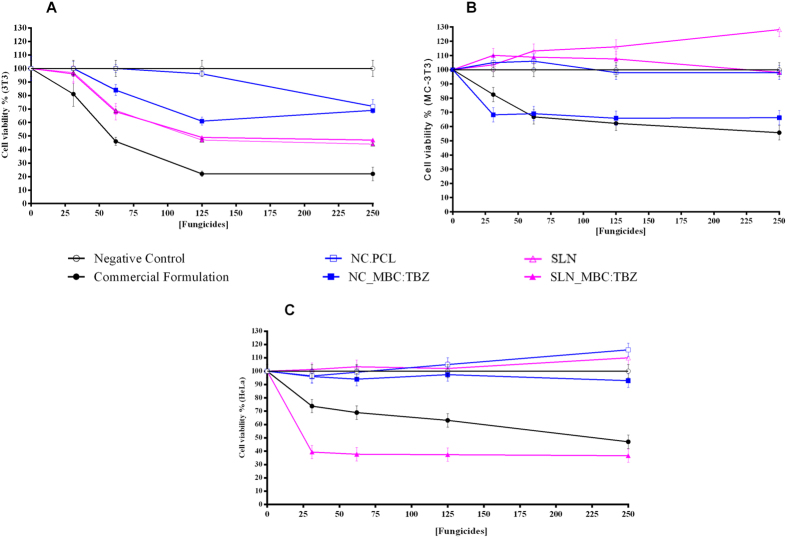
Cytotoxicity evaluation of the different formulations (NC_PCL, NC_MBC_TBZ, SLN, SLN_MBC:TBZ and Commercial Formulation). The fungicide concentrations were investigate in the range of 31.25 and 250 μg/mL. The assays were performed using Balb/c 3T3 mouse fibroblasts (**A**); MC-3T3 osteoblast (**B**) and HeLa adenocarcinoma (**C**) and the MTT reduction test (n = 3). NC_PCL and SLN were tested in the same dilution conditions from the systems containing fungicides.

Apart from the physio-chemical and morphological characterizations, the *in vitro* cellular viability test was conducted to assess the cytotoxicity of the solid lipid nanoparticles loading fungicides (Fig. 5). For this, there different cell types (two normal and one cancerous cell lines) such as preosteoblast (MC3T3-E1); adenocarcinoma (HeLa) and fibroblast (Balb/c 3T3) were employed to evaluate percentage cell viability under the presence of prepared nanoparticles and compared the viability with that of the commercial formulations. The results of the cellular viability assays obtained for normal cells (3T3 and MC3T3) clearly showed that independent of the cell types employed, the current nanoparticles were less toxic than the commercial fungicides (Fig. 5). This can be evidenced from the fact that the percentage cell viability in the presence of newly prepared nanoparticles was lower than that of the commercial product. Moreover, the experimental results indicates that the effect of exposure of the cells to the commercial formulation was dose-dependent, with cellular viability values below 25% and 60% for the cell lines 3T3 and MC-3T3 respectively at the highest concentration, which clearly revealed the cytotoxicity of the fungicides. However, the results obtained with the adenocarcinoma cells (HeLa) showed that the cytotoxicity of the solid lipid nanoparticles loading fungicides was higher than commercial formulation. This result can be attributed to the difference in uptake of these nanoparticles by the cells^43^. Furthermore, it is already known that carbendazim possesses the ability to inhibit the proliferation of human cancer cells^44^,^45^.

**Figure 6:**
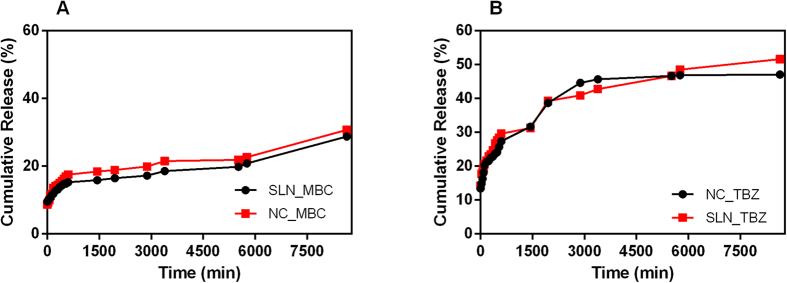
Temporal profiles of fungicide release. Cumulative release of (**A**) MBC and (**B**) TBZ in suspensions of nanoparticles (NCs and SLNs). The tests were performed in triplicate.

As the characterization and the cytotoxicity results indicates good results for these systems, the release kinetics were investigated. The release kinetics can provide important information concerning the interaction between a bioactive compound and a carrier, as well as the mechanism involved in the release process^46^,^47^,^19^. Figure 6 shows the curves obtained for release of the fungicides from the NCs and SLNs. The assays were performed in tube systems, where the nanoparticle formulations were placed in contact with a solvent (deionized water) in order to release the fungicides from the nanoparticles. This enabled the evaluation of the effect of the encapsulation on the rate of release of the fungicides. The release profiles showed that there was substantial release of MBC during the first 600 min of the experiment, after which the release became more gradual. The release profiles of the two nanoparticle formulations were similar, with release of only around 28% and 30% of the MBC after 6 days, for the NCs and SLNs, respectively. These observations provided clear evidence of the capacity of the nanoparticles to modulate the fungicide release profiles.

The release profiles of TBZ contained in the nanoparticle suspensions were initially similar, with rapid release of the fungicide during the first hours of the experiment. Over a period of six days, the polymeric nanoparticles released around 47% of the fungicide, while over the same period the SLNs released around 51%. Hence, the release of TBZ was faster, compared to MBC. These differences in release profiles could be due to the differences in the interactions (such as hydrogen bonds, van der Waals forces, and hydrophobic interactions) between the active agent and the carrier, as well as the greater aqueous solubility of TBZ. It should be pointed out that the duration of the trial was too short for the attainment of 100% release, which would have required a much longer experimental period. In addition, as a quality control measure, the total amounts of the fungicides in the system (considering the free and encapsulated forms) were determined. The values obtained were in agreement with the initial amounts, showing that during the course of the trial there was no microbial degradation or adsorption of the compounds onto the walls of the flask.

**Figure 7:**
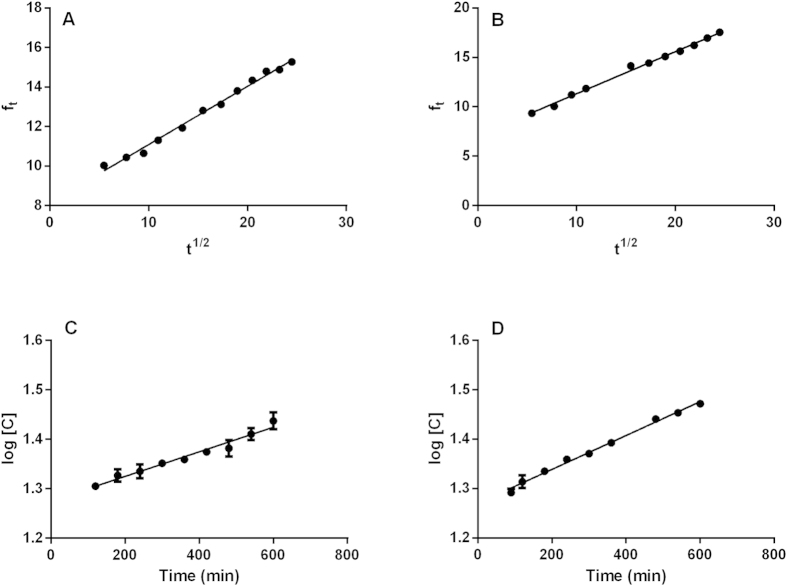
Mathematical analysis of the profiles of release of the fungicides from the nanoparticles. Results obtained for: (**a**) NC-MBC (Higuchi model); (**b**) SLN-MBC (Higuchi model); (**c**) NC-TBZ (first order model); (**d**) SLN-TBZ (first order model).

The release profiles recorded at ambient temperature were analyzed using the Higuchi, first order, and zero order mathematical models (Fig. 7). Linear regression was used to calculate the values of the release exponent (n), the constant (k), and the coefficient (r). The values obtained are listed in Table 1.

The best fits to the profiles of the release of MBC from both types of nanoparticle were obtained using the Higuchi mathematical model. Higuchi kinetics indicates that the release obeys Fick’s Law and proceeds due to simple diffusion^46^,^47^. In the case of TBZ, the best fit was provided by the first order model, which considers that the release of an active agent is time-dependent, with the amount released diminishing over time^47^.

Regardless of the type of carrier system (NCs or SLNs), the experimental results indicated that the release mechanism was more or less similar for each fungicide. However, the different mechanisms observed for the two compounds suggest that there were differences in terms of the interactions between the fungicides and the components of the carrier systems. However, further studies will be required in order to obtain a better understanding of these interactions.

**Figure 8:**
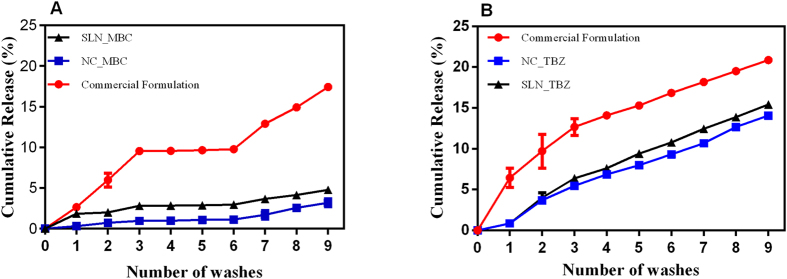
Temporal profiles of fungicide release. Cumulative release of (**A**) MBC and (**B**) TBZ in suspensions of nanoparticles (NCs and SLNs). The tests were performed in triplicate.

In this study the release study of commercial formulation was not realized, because the system employed in this experiment, the suspension it is in direct contact with the solvent (in sink dilution) and in the commercial formulation fungicides is not encapsulated and in this way, we could investigate the release of the fungicides from the commercial formulation as function of time. The comparison between nanoparticles and commercial formulation release was done in soil layer release experiments, because in this system, interactions may occur with the soil layer (Fig. 8).

The use of the nanoparticles significantly reduced the concentration of carbendazim in the leachate (Fig. 8A). After nine washes, there was a release of about 17% of carbendazim from the commercial formulation, while in the case of NC and SLN formulations, the values were found as 3.2 and 4.8%, respectively. The release profiles for tebuconazole (Fig. 8B) also showed that there was a significant decrease in the leaching percentage when the fungicide was associated with the nanoparticles, compared to the commercial formulation. These results demonstrated that loading of the fungicides into the nanoparticles decreased the amounts of the fungicides available for leaching. Interestingly, the release profiles observed in these experiments were analogous to those found in the release assays conducted under sink dilution conditions, with close correlation between the two types of test.

The low release of the fungicides measured for the commercial formulation could be explained by the fact that the compounds have high coefficients of absorption by organic matter (Koc). Molecules with high Koc are fairly insoluble in water, with preferential binding to the soil, and are therefore less likely to experience leaching^48^. Nonetheless, it was clear that the encapsulation acted to reduce the leaching even further. Similar findings have been reported for the herbicide 2,4-D^49^, where its association with polymeric gels (carboxymethylcellulose) decreased leaching. After eight washes, there was 100% release of the free herbicide, while over the same period the use of the gels resulted in less than 80% being released.

The great advantage of possessing a release system that is slow is that the concentration of the active can be adjusted to the minimum effective concentration as a function of time and this system maintains the active concentration in the field whitin the range of biological effectiveness. Thus the possibility of reaching toxic ranges concentrations to non-target organism in the environment is low. Another aspect concerns the issue of energy saving in the field, since there is no need for multiple applications of an asset in function of time, being released slowly, thus generating economic and environmental gains.

**Figure 9:**
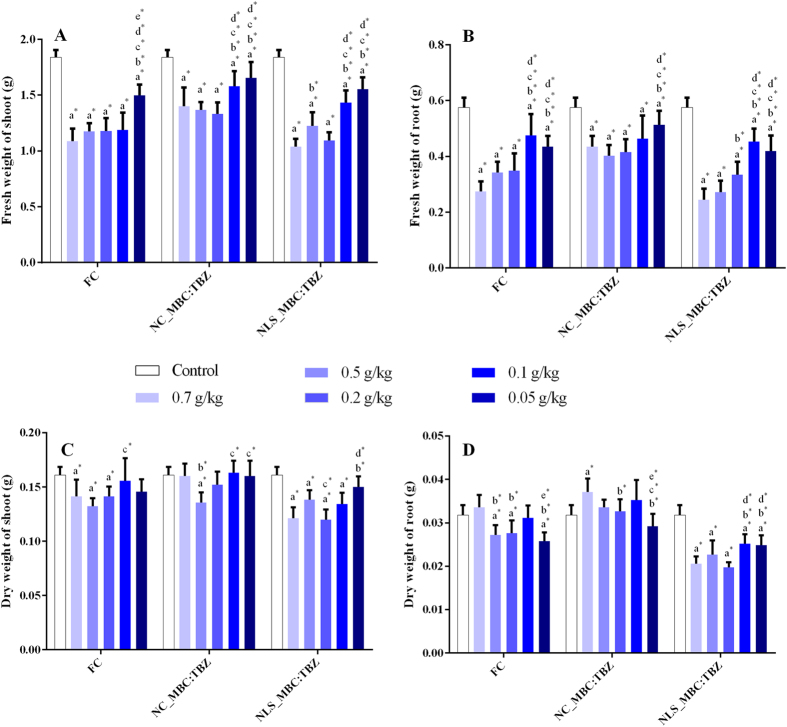
Effect on *P. vulgaris* emergence of treatments using the commercial product (Tebuzin 250 SC) and the nanoparticle formulations at different fungicide concentrations. (**A**) Green mass of the aerial part, 15 days after germination; (**B**) green mass of the root part, 15 days after germination; (**C**) dry mass of the aerial part after drying for 7 days; (**D**) dry mass of the root part after drying for 7 days. The level of significance used was p < 0.05 for the differences observed between the groups, where significant differences are indicated as follows: a* (compared to the control); b* (compared to 0.7 mg/mL); c* (compared to 0.5 mg/mL); d* (compared to 0.2 mg/mL); and e* (compared to 0.1 mg/mL).

The influence of the commercial and nanoparticle formulations on plant emergence was evaluated using *P. vulgaris* seeds. The germination index of the seeds (92%) was not affected by the commercial product or by the nanoparticle formulations (with or without fungicides). However, in the case of emergence, there was a progressive decrease in the fresh weights of roots and shoots when the concentrations of the fungicides were increased (Fig. 9). The fresh weight of the aerial and root parts of the seedlings were analyzed for 15 days after emergence, comparing plants treated using the different formulations with untreated plants. There was a progressive reduction in the biomass of the aerial and root parts when the concentration of the fungicides was increased (Fig. 9A,B). Smaller effects were observed using the PCL nanocapsules containing the two fungicides, compared to the commercial product and the SLN formulation. This could be explained by the more gradual release of the fungicides from the interior of the nanocapsules. The greatest effect on the emergence was observed for the loaded SLNs, for which plant growth was lower than for both the control and the commercial formulation. The results therefore indicated that in terms of fresh plant mass, the loaded NCs caused least impact on plant development, relative to the control. Similar trends were observed for the dry masses of the aerial and root parts of the plants.

Yang *et al*.^39^ also found that reductions in the green masses of the aerial and root parts of maize plants were dependent on the dose of TBZ, and that the mass of the aerial part was more susceptible to suppression by the fungicide, compared to the root part. The use of the nanocapsules therefore seems to offer greater potential for use in agriculture. In this context, the results open new perspectives for the use of sustained release system for these fungicides, which can be more effective in control fungi (to be investigated in future studies) that cause damage to agriculture and reduce de adverse effect to human health and environment.

## Methods

### Nanoparticles preparations

#### PCL nanocapsules

The nanocapsules (NCs) were prepared using the oil-in-water emulsion/solvent evaporation method described by Zhou *et al*.^50^. 10 mg of each fungicide (carbendazim and tebuconazole) were dissolved in 10 mL of acetone containing 200 mg of Myritol, and the resulting solution was added to 20 mL of chloroform containing 400 mg of polymer. This mixture was then sonicated for 1 min at 100 W. The resulting pre-emulsion was added to 50 mL of an aqueous solution containing 150 mg of PVA surfactant and then sonicated for 8 min to form the emulsion. The organic solvent was eliminated and the emulsion was concentrated to a final volume of 10 mL using a rotary evaporator, resulting in a formulation containing MBC and TBZ at concentrations of 1% (m/v).

### Solid lipid nanoparticles

Preparation of the solid lipid nanoparticles (SLNs) was performed according to the emulsification/solvent evaporation method described by Vitorino *et al*.^51^. Firstly, 250 mg of glyceryl tripalmitate and 10 mg (each) of MBC and TBZ were dissolved in 5 mL of chloroform. An aqueous solution (30 mL) was then prepared containing 1.25% (m/v) of PVA. The lipid phase was introduced into the aqueous phase, and the resulting solution was sonicated for 4 min at 40 W. The resulting pre-emulsion was treated by Turrax homogenization at 14000 rpm for 7 min, after which the organic solvent was removed by rotary evaporation and the emulsion was concentrated to a volume of 10 mL. The final concentrations of MBC and TBZ were 1% (m/v).

### Analytical methodology for quantification of the fungicides

The fungicides were quantified by high performance liquid chromatography, using a Varian ProStar system (Agilent Technologies) equipped with an isocratic pump and a UV-Vis detector. The chromatograms were interpreted using Galaxy Workstation software. The analysis of MBC employed a Phenomenex Gemini reverse phase C_18_ column (100 mm × 4.6 mm, 2.6 μm), maintained at ambient temperature. The mobile phase was pure methanol, at a flow rate of 0.4 mL/min, and the detector wavelength was set at 280 nm. Analysis of TBZ employed a Phenomenex Gemini reverse phase C_18_ column (150 mm × 4.6 mm, 5.0 μm), a mobile phase composed of acetonitrile and 1 mM ammonium acetate buffer (60:40, v/v), at a flow rate of 1.4 mL/min, and a detector wavelength of 225 nm. The injection volume was 100 μL. The analytical parameters obtained for MBC were: Y = −6.99592 + 16.15077X, r^2^ = 0.9999, LOD = 0.19 μg/mL, and LOQ = 0.62 μg/mL. For TBZ, the analytical parameters were: Y = −1.74666 + 1.62368X, r^2^ = 0.9994, LOD = 2.79 μg/mL, and LOQ = 9.33 μg/mL.

### Efficiency of encapsulation of the fungicides in the nanoparticles

The total amounts (100%) of the fungicides present in the nanoparticles were determined by completely releasing the compounds by dissolving the nanoparticles in acetone. The resulting solution was filtered through a Millipore membrane (0.22 μm) and the fungicides were determined by HPLC using the analytical conditions described above. The quantities of the fungicides associated with the nanoparticles were determined by the ultrafiltration/centrifugation method, in which the nanoparticle suspensions were centrifuged in ultrafiltration devices composed of regenerated cellulose with a molecular exclusion pore size of 30 kDa (Vivaspin, GE Healthcare). Only the free fungicides were able to traverse the membrane, enabling the amounts associated with the nanoparticles to be calculated as the difference between the total and free concentrations. The measurements were performed in triplicate.

### Characterization of the nanoparticles

#### Nanoparticles stability

Photon correlation spectroscopy was employed to measure the average diameter (hydrodynamic diameter) and polydispersion of the nanoparticles, using a Zetasizer ZS 90 analyzer (Malvern Instruments, UK). Nanoparticle Tracking Analysis (NTA) was performed with a NanoSight LM10 instrument. For DLS measurements, the suspensions of nanoparticles (with and without the fungicides) were diluted in deionized water (1:1000, v/v) and measured at 25 °C, with a fixed angle of 90°. The results were expressed as the averages of three determinations.

The NTA analyses employed a laser with a wavelength of 532 nm (green), a CMOS camera, and NanoSight software (version 2.3). The nanoparticle suspensions were diluted 5,000 times (for the NCs) and 11,000 times (for the SLNs). The analyses were performed in triplicate, with five measurements of approximately 2000 particles per analysis. In order to ensure that replicates of different particles were analyzed, the volume injected into the cell was greater than the cell capacity, hence displacing the previous contents^52^.

### Fourier transform infrared spectroscopy

Infrared spectra were obtained for MBC, TBZ, the PCL polymer, PCL nanocapsules with and without the fungicides, tripalmitin, and SLNs with and without the fungicides. The analyses were performed using a Varian 660 spectrometer equipped with an attenuated total reflectance accessory (GladiATR, Pike Technologies) fitted with a diamond crystal (2.2 mm × 3.0 mm). The instrument was operated in transmittance mode with an angle of incidence of 45^o^, 32 accumulations covering the frequency range from 4000 to 400 cm^−1^, and a resolution of 8 cm^−1^^53^.

### Transmission electron microscopy

Analysis of the morphology and structure of the nanoparticles was performed using a Zeiss LEO 906 microscope operated at a voltage of 80 kV. The nanoparticles obtained immediately after preparation and after 120 days of storage were diluted x1000 in water and then negatively colored. Drops of the nanoparticle suspensions were placed onto 200–300 mesh grids coated with formvar. After drying, drops of an aqueous 2% (m/v) solution of uranyl acetate were added, the solvent was removed by evaporation, and the grids were analyzed.

### Release experiments

The release of the fungicides (MBC and TBZ) from the nanoparticles was evaluated under sink conditions, using a method modified from the procedure described by Asrar *et al*.^34^. A 1 mL volume of the nanoparticle suspension with concentrations of 1% (m/v) of each fungicide was diluted in 625 mL of water. Aliquots (10 mL) of this mixture were placed in closed Falcon tubes and agitated in a shaker (150 rpm) at room temperature. At intervals of 15, 30, and 60 min, over a total period of 8640 min (144 h), one tube of each formulation was removed from the shaker and centrifuged in order to sediment out the nanoparticles. The supernatant was filtered through a 0.45 μm filter. The samples were analyzed using HPLC and the peak area values were converted into percentages of fungicide released as a function of time. The tests were performed in triplicate. The mechanism of release of MBC and TBZ from the nanoparticles was evaluated using the zero order, first order, and Higuchi mathematical models.

The Higuchi model describes a diffusion process that follows Fick’s Law. The release is proportional to the square root of time:


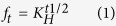


where *f*_*t*_ is the amount of fungicide released at time *t* and *K*_*H*_ is the Higuchi release constant^51^.

The zero order mathematical model (Equation 2) considers the amount of fungicide released in a time *t*. Formulations that exhibit this type of profile release the same amount of compound per unit time, regardless of the amount of compound remaining^54^.


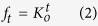


where *f*_*t*_ is the amount of fungicide released at time *t* and *K*_*0*_ is the zero order release constant.

The first order mathematical model (Equation 3) describes release that is dependent on the amount of the compound remaining in the particle. In other words, the amount of compound released decreases over time^54^.


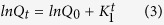


where *Q*_*t*_ is the amount of fungicide released at time *t*, *Q*_*0*_ is the initial amount of fungicide in solution, and *K*_*1*_ is the first order release constant.

### Soil layer release

The leaching of the fungicides (MBC and TBZ) from the nanoparticles and the commercial formulation was evaluated using Buchner funnels. Firstly, 50 g of soil was placed in a Buchner funnel containing a filter paper with pore size of 45 μm. The nanoparticle formulations and the commercial formulation (containing 5 mg of MBC and TBZ) were applied to the soil surface. The funnels were then irrigated with 30 mL of deionized water three times a day, with 2-h intervals between washes. Each treatment was irrigated nine times, in triplicate. The leachate from each wash was collected, filtered through a Millipore membrane (0.22 μm), and the fungicide concentration in the leachate was determined by HPLC^49^. The soil employed in this assay was obtained from a rural forest fragment (latitude 23.432628; longitude −47.369862; 601 m altitude).

### Evaluation of cytotoxicity using the MTT test

In order to assess the cellular viability, assays were performed using the following cellular types: MC3T3-E1, HeLa and Balb/c 3T3. Balb/c 3T3 mouse fibroblasts maintained under continuous culture in DMEM culture medium supplemented with 10% fetal bovine serum, 100 Ul/mL of penicillin, and 100 μL of streptomycin sulfate, at pH 7.4 and a temperature of 37 °C, under a humid atmosphere containing 5% CO_2_. HeLa adenocarcinoma maintained under continuous culture in DMEM culture medium with 1% nonessential amino acids, 2 mM L-glutamine, 1 mM sodium pyruvate, 1500 mg/L sodium bicarbonate and 1 g/L glucose, temperature of 37 °C, under a humid atmosphere containing 5% CO_2_. MC3T3-E1 preosteoblast maintained under continuous culture in Alhpa Minimum Essential medium with ribonucleosides, desoxyribonucleosides, 2 mM of L-glutamine and 1 mM of sodium pyruvate, without ascorbic acid, temperature of 37 °C, under a humid atmosphere containing 5% CO_2_.

Plating out was performed by inoculating 1 × 10^4^ viable cells into 96-well plates, followed by incubation for 48 h until semi-confluence was reached. The cells were then exposed for 24 h to the commercial formulation and the nanoparticles containing the fungicides at concentrations ranging from 31.25 to 250 μg/mL. Cellular viability was determined using the reduction of 3(4–5 dimethylthiazol-2-yl)-2,5-diphenyltetrazolium bromide (MTT). The cells were incubated with MTT (0.5 mg/mL) for 3 h at 37 °C, after which the number of viable cells was determined by measuring the amount of MTT converted to formazan (a purple compound) by the mitochondrial dehydrogenases. The formazan crystals were dissolved in ethanol by agitation for 30 min at ambient temperature. The absorbance of the solution in each well was then measured at 570 nm using a plate reader (Multiskan MS, Labsystems) and the percentages of viable cells were calculated^55^,^56^.

### Plant emergence assays

Seeds of Phaseolus vulgaris were treated with the commercial formulation (Tebuzim 250 SC) and with the nanoparticle formulations containing the fungicides. A control treatment was performed using water alone. The concentrations tested were equivalent to 0.05, 0.1, 0.2, 0.5, and 0.7 g fungicide/kg of seeds. The seeds were shaken with the formulations manually in plastic bags for 60 s, and then left in the dark for 24 h to allow total absorption of the solutions. The seeds were subsequently planted in trays containing growth substrate (Hortaliças Mix). Fifteen days after sowing, the plants were collected, washed to remove the substrate, and divided into aerial and root sections that were weighed to determine the green mass. The dry mass was measured after drying the plants in a drying cabinet at 37 °C for 7 days. All measurements were performed in triplicate, using three plants per replicate^39^. Statistical analysis of the data was performed as described above.

## Additional Information

**How to cite this article**: Campos, E. V. R. *et al*. Polymeric and Solid Lipid Nanoparticles for Sustained Release of Carbendazim and Tebuconazole in Agricultural Applications. *Sci. Rep*. **5**, 13809; doi: 10.1038/srep13809 (2015).

## Figures and Tables

**Table 1:** Results obtained for the fits of the different mathematical models.

Parameters	Higuchi	First order	Zero order
NC_MBC			
Release constant (k)	**8.14** **min**^−**1**^	3.7 × 10^−3^ min^−1^	0.014 min^−1^
Correlation coefficient (r)	**0.9921**	0.9771	0.9888
SLN_MBC			
Release constant (k)	**7.10** **min**^−**1**^	4.9 × 10^−3^ min^−1^	0.014 min^−1^
Correlation coefficient (r)	**0.9948**	0.8879	0.9751
NC_TBZ			
Release constant (k)	0.543 min^−1^	**2.4** × **10**^−**3**^** min**^−**1**^	15.7 min^−1^
Correlation coefficient (r)	0.9470	**0.9865**	0.9046
SLN_TBZ			
Release constant (k)	0.478 min^−1^	**3.4** × **10**^−**3**^ **min**^−**1**^	16.8 min^−1^
Correlation coefficient (r)	0.9913	**0.9936**	0.9771
